# Removal of Sulfadiazine by Polyamide Nanofiltration Membranes: Measurement, Modeling, and Mechanisms

**DOI:** 10.3390/membranes11020104

**Published:** 2021-02-02

**Authors:** Haochen Zhu, Bo Hu, Fengrui Yang

**Affiliations:** 1 State Key Laboratory of Pollution Control and Resources Reuse, Key Laboratory of Yangtze River Water Environment, College of Environmental Science and Engineering, Tongji University, Ministry of Education, 1239 Siping Rd., Shanghai 200092, China; 1851535@tongji.edu.cn (B.H.); andriy02121804@163.com (F.Y.); 2 Shanghai Institute of Pollution Control and Ecological Security, Shanghai 200092, China

**Keywords:** sulfadiazine, rejection rate, nanofiltration membrane, polyamide, modeling

## Abstract

In this study, a complete steric, electrostatic, and dielectric mass transfer model is applied to investigate the separation mechanism of typical antibiotic sulfadiazine by NF90, NF270, VNF-8040 and TMN20H-400 nanofiltration membranes. FTIR and XPS analysis clearly indicate that the membranes we used possess skin layers containing both amine and carboxylic acid groups that can be distributed in an inhomogeneous fashion, leading to a bipolar fixed charge distribution. We compare the theoretical and experimental rejection rate of the sulfadiazine as a function of the pressure difference across the nanopore for the four polyamide membranes of inhomogeneously charged nanopores. It is shown that the rejection rate of sulfadiazine obtained by the solute transport model has similar qualitative results with that of experiments and follows the sequence: $R_{NF90}>R_{VNF2-8040}>R_{NF270}>R_{TMN20H-400}$. The physical explanation can be attributed to the influence of the inhomogeneous charge distribution on the electric field that arises spontaneously so as to maintain the electroneutrality within the nanopore.

## 1. Introduction

Antibiotics are organic substances which organisms (including microorganisms, plants and animals) produce or otherwise acquire in the course of their life activities that can selectively inhibit or affect other biological functions at low concentrations [1]. Sulfonamides (SAs) are a kind of common antibiotic, which are widely used in medical treatment, aquaculture and animal husbandry. Among most consumed SAs, sulfadiazine (SDZ) is employed in the cure of mild-to-moderate infections not only in human, but also in veterinary therapies. The concentration of sulfadiazine in sewage treatment plants or water environment is usually ng/L or μg/L, but the concentration of sulfadiazine in liquid fertilizer or landfill leachate can reach 1–20 mg/L. However, due to the incompletely metabolized in organisms, most of sulfadiazine is discharged directly into soil and water in the form of antibiotic matrix and metabolites, and finally returns to the aquatic environment [2]. In addition, SDZ cannot be effectively removed via traditional water treatment technology, owing to its biological toxicity and accumulation. The rising membrane technologies such as ultrafiltration (UF), nanofiltration (NF) and reverse osmosis (RO) have obvious advantages for removal of trace organic pollutants such as antibiotics [3,4,5].

Among all, nanofiltration is the most recently developed pressure-driven membrane process for liquid-phase separations. The molecular weight cut off (MWCO) of NF membranes is in the range 100–1000 Da and their pore size is expected to be of the order of the nanometer. NF membranes can thus treat solutions containing multivalent ions and organic compounds with very low molecular weight at a relatively low operation pressure [6,7,8]. This results from the combination of pore diameters of a few nanometers with electrically charged materials.

At present, thin film composite membranes (TFM) prepared by interfacial polymerization (IP) methods occupy most of the NF market. The membranes possess a fixed charge that results from dissociation of surface groups (such as, e.g., amine or carboxylic acids) when they are in contact with polar media and/or by adsorption of charged species [9]. Since NF membranes have pore sizes of the same order of magnitude as the distances of action of long-range forces, exclusion mechanisms are affected not only by steric effects but also by electrostatic interactions. The latter is known as the Donnan exclusion, i.e., the exclusion mechanism resulting from the Coulomb interaction between charged solutes and the membrane fixed charge [10]. Hence, for an uncharged trace organic the rejection performance is governed by steric exclusion, while the rejection of polar trace organics are significantly affected by both steric and Donnan exclusions between organic molecules and charged membrane surface [11]. Yeomin investigated the removal of EDC/PPCPs of 27 compounds by nanofiltration (NF) membranes by considering the influence of membrane pore size, molecular polarity and surface fixed charge. Results showed that the fixed charge of membrane and the molecular size of compounds were the main factors affecting the retention performance of nanofiltration membrane [12]. 

Indeed, the fixed charge on the surface of the nanofiltration membrane determines the separation performance of the nanofiltration membrane for charged particles, and the modification of the membrane surface with electrical properties can achieve higher retention performance. On the one hand, it is related to the physical and chemical properties and operational parameters of solute, such as the molecular weight and valence state of solute, the concentration of inlet liquid, the flow rate and the pressure. On the other hand, the electrostatic separation performance of nanofiltration is related to the charge properties on the membrane surface. To this end, Shah et al. have systematically evaluated the mechanisms of antibiotic removal by commercial polyamide nanofiltration membranes and predicted their rejection by the Donnan steric partitioning model. Results show that the rejection of carbadox calculated by the model is underestimated relative to the experimental findings between pH 7 and 9 [13]. To our knowledge, this is largely due to the fact that the bipolar characteristics of the polyamide nanofiltration membranes are not considered in the model. As mentioned previous, polyamide membranes obtained by interfacial polymerization possess skin layers containing both amine and carboxylic acid groups that are distributed in an inhomogeneous fashion, leading to a bipolar fixed charge distribution. The inhomogeneity of the active layer on the polyamide nanofiltration membrane is mainly caused by reactor time, monomers concentration, curing temperature and other factors during the synthesis. Indeed, through the direct observation of the inner structure of different polyamide membranes by advanced material analysis technology such as transmission electron microscopy (TEM), Freger has indicated that an external negative region is located on top of an internal region containing a positive charge [9]. Ouyuan et al. prepared a bipolar charged nanofiltration membrane with an active skin layer by modifying the polyether sulfone ultrafiltration membrane of a positively charged quaternate chitosan (HTCC) and a negative charged polydopamine (PDA). Experimental results showed that an excellent rejection rate has obtained on the removal of atenolol (ATE), carbamazepine (CBZ) and ibuprofen (IBU) due to the inhomogeneous distributions of fixed charge on the membrane surface [14]. The physical phenomenon involved in the separation performance is mainly attributed to the electric field that arises spontaneously due to the non-uniform charge distribution along the nanopore, which limits the transport of the antibiotics and thus increases the rejection performance.

Another important exclusion mechanism of NF is based on the dielectric phenomenon which is connected with the difference between the dielectric constant of the pore-filling solution ($\varepsilon_{p}$) and that of the (external) bulk solution ($\varepsilon_{b}$) (Born effect) proposed by Yaroshchuk [15,16]. The so called Born effect is due to the variation of solvation energy (i.e., the work of charge transfer) resulting from the difference in dielectric constant of solution between outside and inside pores. Numerous studies have shown that the Born effect in dielectric phenomena is likely to play a significant role in NF [17,18,19,20]. However, few studies have considered dielectric effects in conjunction with the bipolar characteristics of the polyamide membranes [21,22]. The major reason is that accounting for the dielectric exclusion mechanism inevitably increases the number of fitting parameters, making the use of current NF models less appropriate for predictive purposes [23]. 

In this work, a complete steric, electrostatic, and dielectric mass transfer-model (SEDE) will be responsible for elucidation of the nanofiltration separation mechanism of typical antibiotic sulfadiazine. Four bipolar polyamide membranes with different physical and chemical properties will be used to reject sulfadiazine respectively. The experimental results are compared with those of SEDE mass transfer model, and the mechanism of sulfadiazine retention is clarified by analyzing the variation of electric field intensity and ion distribution in the nanopore channel. 

## 2. Materials and Methods

### 2.1. Membranes and Chemicals

Four commercial polyamide nanofiltration membranes are tested, namely NF90, NF270 supplied by Filmtec Dow, VNF2-8040 obtained from VONTRON and TMN20H-400 provided by TORAYFIL. The characteristics of each membrane are listed in Table 1. All four nanofiltration membranes used for this research are composed of an extremely thin polyamide active layer, which identifies the membrane separation performance. Despite the similar composition in these active layers, the accurate polymeric constitutions are indistinct and deemed to be quite different. Accordingly, these membranes are distinct comparatively from one another in their features. 

Standard Sulfadiazine (C_10_H_10_N_4_O_2_S, >98.0%) was purchased from Ourchem Co., Ltd. (Shanghai, China). The physico-chemical properties of SDZ are listed in Table 2. pKa1 and pKa2 demonstrate the ampholytic characteristic of sulfadiazine as described by the ionization equilibria. It exists thus in various forms in water and some sulfadiazine gives protons forming a negative charge of -1 when pH=7 (see the percent speciation in Table 2). The diffusion coefficient ($D_{s}=6.14\times 10^{-10}\;m^{2}/s$) was obtained from the Drugbank database and stokes radius ($r_{s}=0.4\;\mathrm{nm}$) was calculated from the Stokes–Einstein equation [26]. Sulfadiazine was dissolved in pure acetonitrile with deionized (DI) water (19.1 Ω/cm, HITECH Prima-Q15 purification system) by ultrasound. The stock solutions (1 mg/L) were stored in a refrigerator at 0–4 °C. Trace amounts of 1 mol/L NaOH or HCl (Ourchem Co., Ltd., China) were used to adjust the initial pH of the solution.

### 2.2. Membrane Pore Size Determination

Dioxane, erythritol and xylose were used as organic tracers to characterize the average pore size of the four commercial polyamide NF membranes. These organic solutes have low molecular weight, neutral charge, inert and do not adsorb on the membrane. The physicochemical parameters of the selected organic tracers are shown in Table 3. All organic tracers were analyzed by a UV-VIS Benchtop spectrophotometer (DR 6000, Hach, Loveland, CO, USA) and purchased from Ourchem Co., Ltd. (China).

### 2.3. Experiment System and Operations

All experiments were performed in a lab-scale crossflow SEPA–CFII test cell (Sterlitech Co., Ltd. Kent, WA, USA) with effective membrane area of 140 cm^2^. Both the retentate and permeate were circulated into the feed container for the purpose of maintaining concentration of feed solution constant. Before each experiment, the NF membrane was immersed in DI water for at least overnight to ensure complete wetting. The membrane was pre-compacted with DI water at the operating pressure of 2 MPa and flow rate of 2 LPM for at least 2 h in order to avoid compression effects [30]. Permeation experiments of sulfadiazine solution were then carried out with the flow rate of 2 LPM and feed pressure varied between 0.4 and 2 MPa. All cross-flow filtrations were executed under the constant temperature of 25 °C with a cooling circulation system. 

### 2.4. Analytical Methods

Concentrations of the SDZ in feed and permeate were analyzed by a high-performance liquid chromatography (HPLC, Agilent 1200, LA, USA) equipped with a UV/VIS diode array detector and a C184296 column (XBridge 250 × 4.6 mm^2^, 5 μm). The detection wavelength of SDZ absorption was set at 223 nm. The mobile phase consisted of 75% acetic acid as solvent A and 25% acetic acid as solvent B at a flow rate of 1.2 mL/min. The final injection volume of 50 μL was also used and the column temperature was set at 35 °C. The retention time of SDZ was about 2.6 min. All calibrations were linear with coefficients of determination (R2) at least 0.99 by applying the external standard method.

### 2.5. Membrane Characterization

Chemical structures of the membranes were characterized with Attenuated total reflection Fourier transform infrared spectroscopy (ATIR-FTIR, Nicolet Nexus 470, LA, USA) in order to analyze the similarities and differences of different nanofiltration membranes in materials. All samples were dried completely in vacuum at 25 °C for 24 h prior to measurement. The elemental information of membrane surface and the chemical analysis were characterized by X-ray photoelectron spectroscopy/electron spectroscopy (XPS, Thermo ESCALAB 250XI, US). Spectral measurements were run in the binding energy range of 0–1000 eV, collected by high resolution (0.1 eV) scans of the C ls, O 1s, N 1s and S 1s with pass energy of 50 eV. To catch the electrokinetic phenomenon of membrane surface charge, zeta potentials (ζ) were measured by the tangential streaming potential (TSP) technique using a SurPASS™ 3 electrokinetic analyzer (Anton Paar, Graz, Austria). KCl solution was used as a background electrolyte of 1 mM to determine the zeta potential values at room temperature (25 ± 0.5 °C.). The pH was adjusted from 3 to 9 by addition of 0.1 M HCl and KOH solutions and measured using a pH-meter.

### 2.6. Solute Transport Model

**Figure 1:**
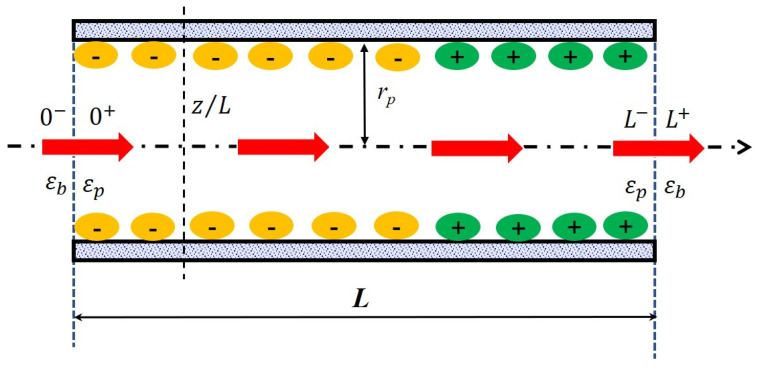
Principle scheme of the nanopore applied in the solute transport modeling.

In order to catch all processes involved in a separation at the nanometer scale, a multi-physic modeling coupling the hydrodynamics of the system with fluid/material interactions (steric hindrance, electrostatic interactions, dielectric phenomena…) has been carried out. Within the scope of the steric, electric and dielectric exclusion (SEDE) model [31], the active layer of the membrane is described as a bundle of straight cylindrical pores of length L and radius *r_p_* (with $r_{p}\ll ∆L$ so that edge effects can be neglected) separating the feed solution from the permeate one (see Figure 1). ∆w corresponds to the effective thickness of the active layer and so, it implicitly includes the tortuosity factor of the real membrane. The solution/membrane interface and the membrane/solution interface are marked as “0^−^|0^+^” and “L^−^| L^+^” to present the inlet and outlet of a pores. The arrow across the pore means the direction of flow. The position inside the pore is marked as “x/L”. The bulk solutions are assumed to be ideal and extremely stirred therefore the polarization phenomena at the membrane surface is not considered in this work. 

The extended Nernst–Planck equation (ENP) forms the basis for the description of solute transport through charged porous membranes and reads as follows,

(1) $$j_{i}=-K_{i,d}D_{i,\infty }\frac{dc_{i}}{dx}-\frac{z_{i}c_{i}K_{i,d}D_{i,\infty }}{RT}F\frac{d\psi }{dx}+K_{ic}c_{i}\frac{J_{v}}{A_{k}}\;\left(x=\frac{z}{L},\;0<x<1\right)$$

with *j_i_* the molar flux of solute *i* through pores, *J_v_* is the permeate volume flux, *D_i_*_,∞_ its diffusion coefficient at infinite dilution, *ψ* the local electric potential inside pores, *V* the solvent velocity inside pores, *K_i*,*d_* the hindrance factor for diffusion inside pores and *K_i*,*c_* the factor accounting for the effect of pore walls on the solute convective flux. *A_k_* is the membrane porosity, *F* is the Faraday constant, *R* is the ideal gas constant, *T* is the temperature, φ denotes the local electric potential inside pores.

Within the scope of the uniform potential approximation [32], the concentration gradient of solute and electrostatic potential inside the nanopore are defined as radially averaged quantities and the ENP equation (Equation (1)) can be rewritten as follow,

(2) $$\frac{dc_{i}}{dx}=\frac{J_{V}}{K_{i,d}D_{i,\infty }A_{k}}\left(K_{i,c}c_{i}-c_{i}\left(L^{+}\right)\right)-\frac{z_{i}Fc_{i}}{RT}\frac{d\psi }{dx}$$

The local electric potential inside pores is regulated by the Poisson equation,

(3) $$\nabla^{2}\psi =-\frac{F}{\varepsilon_{0}\varepsilon_{r}}\underset{i}{\sum }c_{i}z_{i}$$

where $\varepsilon_{0}$ is the vacuum permittivity and $\varepsilon_{r}$ is the solution dielectric constant. In the uniform potential approximation, the Poisson equation is no longer considered and has to be coupled with an explicit expression of the local electroneutrality inside the nanopore,

(4) $$\overset{n}{\underset{i=1}{\sum }}z_{i}c_{i}\left(x\right)+C_{Loc}\left(x\right)=0\;\left(0<x<1\right)$$

where $C_{Loc}$ represents the local fixed charge concentration of the nanopore which is connected with the surface charge density (*σ*) of a cylindrical pore by

(5) $$C_{Loc}\left(x\right)=\frac{2\sigma \left(x\right)}{Fr_{p}}$$

Considering that the charge of the organic membranes may depend on the properties of salt and ionic strength, the surface charge density can be described by zeta potential (ξ) according to the Gouy–Chapman theory [33]:

(6) $$\sigma =-sign\left(\zeta \right)\sqrt{2\varepsilon_{0}\varepsilon_{b}RT\underset{i}{\sum }c_{i}^{bulk}\left[exp\left(-\frac{z_{i}F}{RT}\zeta \right)-1\right]}$$

where $c_{i}^{bulk}$ is the concentration of solute *i* in the feed solution.

It must be emphasized that formula (6) can only roughly estimate the surface charge density of nanopore as it doesn’t consider charge regulation. This implies that the surface potential inside the nanopore is supposed to be identical with that of outer membrane surface and σcan only be deemed to as an overestimation of the surface charge density [34].

The axial electric field along the pore length can be derived from Equations (2) and (5) and expressed as follows,

(7) $$E\left(x\right)=-\frac{d\psi \left(x\right)}{d\left(x\right)}=-\frac{\sum_{i}\left(\frac{z_{i}J_{v}}{K_{i,d}D_{i,\infty }A_{k}}\right)\left(K_{i,c}c_{i}\left(x\right)-c_{i}\left(L^{+}\right)\right)}{\left(\frac{F}{RT}\right)\sum_{i}c_{i}\left(x\right)z_{i}^{2}}-\frac{\left(\frac{dC_{Loc}\left(x\right)}{d\left(x\right)}\right)}{\left(\frac{F}{RT}\right)\sum_{i}c_{i}\left(x\right)z_{i}^{2}}$$

The distribution of solutes at the membrane/solution interfaces is described by the following partitioning equations (Equations (8) and (9)) including steric, electric and dielectric exclusion,

(8) $$\frac{c_{i},\left(0^{+}\right)}{c_{i},\left(0^{-}\right)}=\varphi_{i}exp\left(-z_{i}∆\psi_{D,\left(0^{+}|0^{-}\right)}\right)exp\left(-∆W_{i,Born}\right)\;$$

(9) $$\frac{c_{i},\left(L^{-}\right)}{c_{i},\left(L^{+}\right)}=\varphi_{i}exp\left(-z_{i}∆\psi_{D,\left(L^{-}|L^{+}\right)}\right)exp\left(-∆W_{i,Born}\right)\;\;$$

where $\varphi_{i}$(= (1 − $r_{i,\mathrm{Stokes}}/r_{p}$)^2^) is the steric partitioning coefficient for solute *i* which is defined as the ratio between the available section for a solute *i* (i.e., taking into account the zone inside the pore in which the ion center cannot penetrate because of its finite size) and the pore cross section [35]. Note that this steric approach proposed by Ferry is only valid when the size of solute is lower than that of pores. The symbol ∆ denotes a variation with respect to the bulk, $∆\psi_{D}$ is the normalized Donnan potential and ∆*W*’*_i*,*Born_* is the solvation energy variations due to the Born dielectric effect described as follow,

(10) $$∆W_{i,\;Born}=\frac{{\left(z_{i}e\right)}^{2}}{8\pi \varepsilon_{0}kTr_{i,cav}}\left(\frac{1}{\varepsilon_{p}}-\frac{1}{\varepsilon_{b}}\right)$$

where $\varepsilon_{p}$ and $\varepsilon_{b}$ represent the dielectric constant of solution inside the pore and bulk solution, respectively. The Born dielectric effect arises if the dielectric constant of the solution varies when the solution enters the membrane pores (due to confinement). The variation of solvation energy (i.e., the work of charge transfer) resulting from the difference in dielectric constant between external and internal (i.e., inside pores) solutions can be estimated on the basis of the Born model [36]. 

Finally, the separation properties of the membrane are quantified by computing the solute rejection *R_i_* defined by:

(11) $$R_{i}=1-\frac{c_{i},\left(0^{-}\right)}{c_{i},\left(L^{+}\right)}$$

## 3. Results and Discussion

### 3.1. ATR-FTIR Analysis

**Figure 2:**
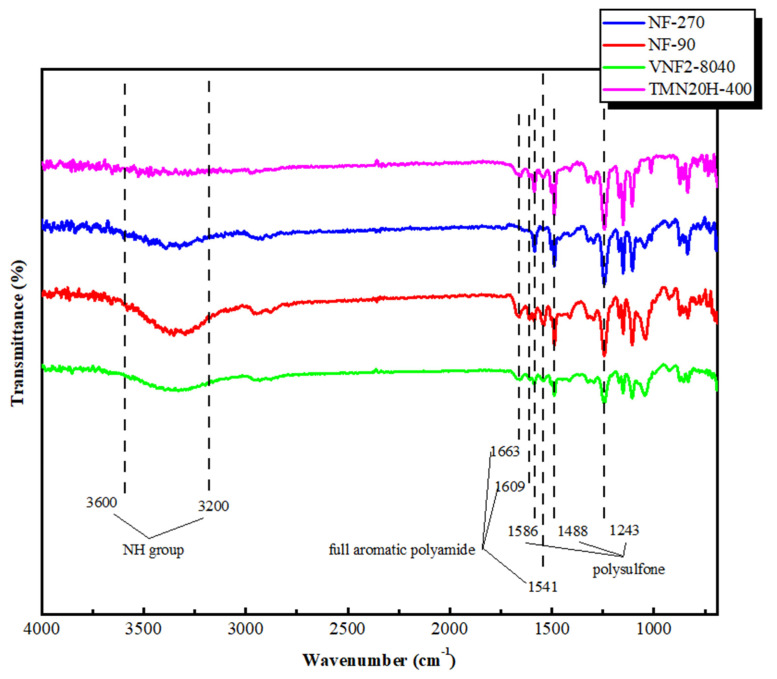
ATR-FTIR spectra of NF90, NF270, VNF2-8040 and TMN20H-400 membranes, respectively.

The chemical composition of membrane surfaces was characterized by employing the typical ATR-FTIR spectra that shown in Figure 2. For all the membrane investigated, the aromatic bands at 1586 and 1488 cm^−1^ were found and these two picks were due to C=C stretching vibration of the aromatic ring of support layer that were specific for polysulfone (PSF) [37,38,39]. The bonds at 1327–1293 cm^−1^ and 1178–1147 cm^−1^ were attributed to the symmetric and asymmetric S=O stretching vibrations of the PSF [40,41,42]. Similar peaks at 1663, 1609 and 1541 cm^−1^ were observed for the classic NF90 nanofiltration membrane and the two new commercial membranes (VNF2-8040 and TMN20H-400). These picks correspond to the fully aromatic polyamide (PA) membranes synthesized by 1,3-benzenediamine (MPD) and trimesoyl chloride (TMC). Among these, the amide I band at 1663 cm^−1^ was related to the C=O stretching, C–N stretching, and C–C–N deformation vibration in a secondary amide group [41]. The aromatic amide was positioned at 1609 cm^−1^ and had been associated with N–H deformation vibration and C=C ring stretching vibration [41]. The peak at 1541 cm^−1^(amide II band) was assigned to the N–H in-plane bending and N–C stretching vibration of a –CO–NH-group [43]. Nevertheless, these peaks were disappeared for NF270, since it is composed of semi-aromatic poly-piperazinamide [44]. The FTIR spectra in the high wave number region (3600–3200 cm^−1^) was related to the stretching of the NH group of the peptide bond of primary amide in the solid state [45]. The FTIR spectra of the four commercial membranes clearly showed that they contained important functional groups, such as OH, NH and CO. Strong bands in the region 3600–3200 cm^−1^ and 1600–1300 cm^−1^ demonstrated the presence of aromatic and heteroaromatic structures as the support and active layer displayed this type of structure in their polymeric chain.

### 3.2. XPS Analysis

**Figure 3:**
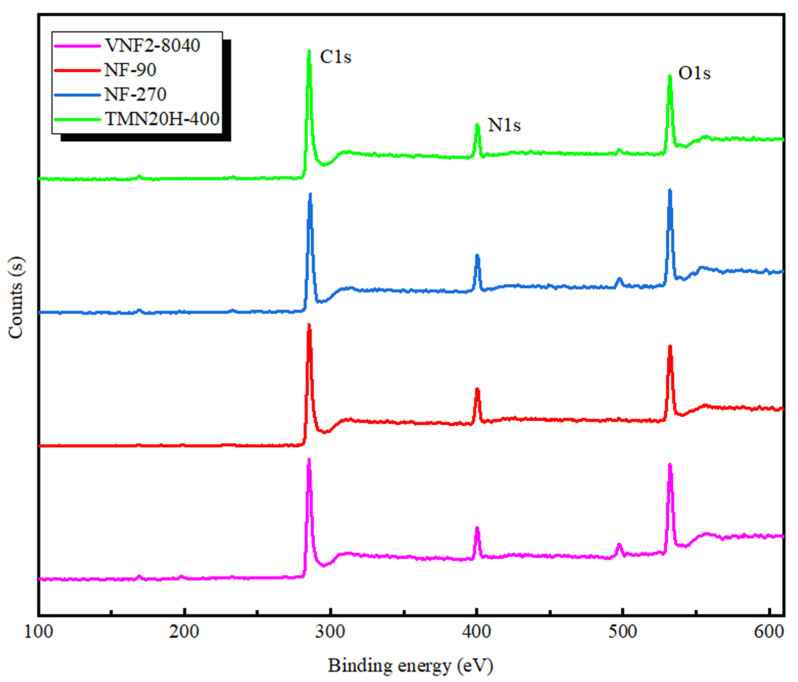
XPS spectra of NF90, NF270, VNF2-8040 and TMN20H-400 membranes, respectively.

In order to further investigate the chemical composition of all the membranes, XPS analysis was applied and the survey spectra are plotted in Figure 3. According to the survey spectra shown in Figure 3, all the membrane surfaces mainly included carbon, oxygen and nitrogen which were identified based on the intensity of the C1s, O1s and N1s peaks that located around 284, 532 and 399 eV, respectively. The analysis of surface elemental compositions is listed in Table 3. It is found that the atomic ratios of oxygen to nitrogen for all the membranes varies between 1 and 2, which corresponds to a fully crossed-linked polyamide (i.e., each oxygen atom is conjoined to a nitrogen atom in an amide bond) and a fully linear with excess oxygen atoms in the free carboxylic groups, respectively [41]. For the fully aromatic PA membranes based on the TMC and MPD (NF90, VNF2-8040 and TMN20H-400), the corresponding carbon contents of fully crossed-linked polyamide (C_6_H_4_ON) and fully linear (C_15_H_10_O_4_N_2_) is 75.0% and 71.4%, respectively [46]. As shown in Table 3, membranes NF90 (O/N = 1.17, C% = 73.2) and VNF2-8040 (O/N = 1.25, C% = 72.45) had relatively low O/N ratios and high carbon content, while the ratio O/N of TMN20H-400 was close to 2 and had a lower carbon content of 68.4%. These results indicate that NF90 and VNF2-8040 can be regard as fully crossed-linked polyamide and membrane TMN20H-400 is a fully linear material. For membrane NF270, we find that the carbon content is 69.92% and the ratio O/N = 1.35 which can be strictly stood for a semi aromatic chemistry with highly crossed-link density (for membrane based on the piperazinamide, a fully crossed-linked membrane has molecular formula C_5_H_5_ON with C% = 71.4% and O/N = 1 and a fully linear one have molecular formula C_12_H_12_O_4_N_2_ with C% = 68.4% and O/N = 2) [41]). This is also consistent well with the absence of characteristic peaks of fully aromatic in its FTIR spectra. 

As a result, FTIR and XPS analysis clearly indicate that these four membranes possess skin layers containing both amine and carboxylic acid groups that can be distributed in an inhomogeneous fashion, leading to a bipolar fixed charge distribution.

### 3.3. NF Membrane Pore Size Estimation

According to the Liu et al. [47], the average pore size of NF membrane is estimated by the obtained real retentions applying the solute transport model presented previously. The ratio of the solute radius to the membrane pore radius $\lambda =r_{s}/r_{p\;}$ is the most momentous model parameter, which defines the steric partition factor by $\varphi_{i}$ and the hindrance coefficients *K_i*,*d_* and *K_i*,*c_*. Several approximate analytical expressions of *K_i*,*d_* and *K_i*,*c_*. derived using the centerline approximation are available in the literature [35]. In this work, we have used the approximate equations derived by Bungay and Brenner [48] because they are applicable over the entire range of the solute-to-pore size ratio [49]. At very high permeate fluxes, the parameter $K_{i,c}=\frac{\left(3-{\varphi_{i}}^{2}\right)\left(\frac{{\left(1-\lambda_{i}\right)}^{2}}{3}\right)}{2}$ is a function of the variable λ and thus is applied to determiner λ for each solute and membrane. With the value of λ and the given solute radius $r_{s}$, the membrane average pore radius is computed for each neutral organic matter retention data as shown in Table 4.

As shown in Table 4, for the classic NF90 and NF270 membrane, our measurements of the pore size are in good agreement with the results obtained in the literature [24,37]. The order of the membrane pore size is as follow: NF90 ≈ VNF-8040 < NF270 < TMN20H-400. The NF90 and VNF-8040 are relatively tight nanofiltration membrane with average pore radius of 0.34 nm and 0.35 nm, respectively. As mentioned in Part 2.6, the steric approach is valid as the pore size is larger than that of solute, and thus electrostatic and dielectric effects are taken into account only for the two membranes. Indeed, this neglect will cause some deviation in the simulation results, but compared with the other two effects, it will not have a significant impact on the calculation of the rejection rate. In contrast, the NF270 and TMN20H-400 can be regarded as a loose nanofiltration membrane with the pore radius of 0.42 nm and 0.44 nm, respectively. These conclusions are consistent with the material characteristics analysis obtained in XPS, which demonstrate a fully linear material for TMN20H-400 and a semi aromatic chemistry for NF270. Indeed, the average pore size of the NF270 and TMN20H-400 are relatively larger than the stokes radius of the sulfadiazine ($r_{s}=0.4\;\mathrm{nm}$) examined in this study, which signifies that steric effect has the least influence on its rejection efficiency and thus the effect of electrostatic and dielectric exclusion dominates the membrane separation performance. 

### 3.4. Pore Dielectric Constant Calculation

The dielectric constant of aqueous solution confined in NF membranes exhibits a strong dependence on the shape and the size of the enclosure. Indeed, although in principle the dielectric constant inside pores could be determined from dielectric relaxation measurements, in practice, it is extremely difficult to determine this parameter because NF membranes are multilayer composite materials. Therefore, numerous researches on the properties of dielectric constant inside the nanoconfined pore are carried out by means of computer simulations. To this end, Senapati and Chandra [50] investigated the dielectric properties of water confined in a spherical nanocavity by applying molecular dynamics simulations. It was found that the dielectric constant of water decreased by nearly 50% under 1 nm pore diameter and the decay rate of the dielectric constant value was linearly related to the size of the pore diameter that can be described as follow,

(12) $$\varepsilon_{p}=\frac{24.904^{0.043r_{p}}}{2}$$

Combined with the calculation results of the pore size of the membrane, the dielectric constants corresponding to different membranes are listed in Table 4.

It must be stressed that the dielectric constant of the solution confined inside the membrane pores is used as a fitting parameter in all current NF models [31,51,52] and exhibits contradictory results that predicting either a decrease or an increase in the dielectric constant of the solution confined inside pores with respect to its bulk value [31,53]. This argument is mainly intuitive since no experimental measurement of the dielectric constant inside pores of NF membranes have been reported yet. However, it is currently believed that confinement should decrease dielectric constant because solvent molecules in such environments are expected to exhibit a greater degree of spatial and orientational order.

### 3.5. Surface Fixed Charge Analysis

**Figure 4:**
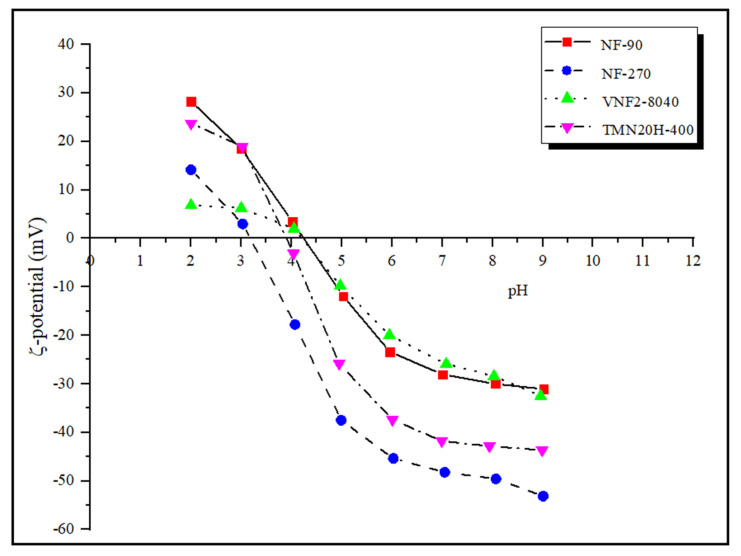
Zeta potentials of of the four commercial nanofiltration membranes in the pH range between 2 and 9.

The charge characteristics of the membrane surface is determined by zeta potential measured by the tangential streaming potential method. In general, a surface charge can be induced on the polyamide nanofiltration membranes by dissociation of functional groups (such as the carboxylic acid and amine) when they are in contact with polar media and/or by adsorption of charged species. The zeta potential under strong acid and strong base conditions reflects the total amount of amine ($-{\mathrm{NH}}_{2}\to -{\mathrm{NH}}^{3+}$) and carboxyl groups ($-\mathrm{COOH}\to -{\mathrm{COO}}^{-}$) on the membrane surface. Figure 4 presents the zeta potentials of the four commercial nanofiltration membranes in the pH range between 2 and 9. It can be seen that the number of amine groups on different membrane surfaces at pH = 2 follows the series: NF90 > TMN20H-400 > NF270 > VNF2-8040 while under strong base condition (pH = 9) the number of carboxyl groups of all the four membrane follows the series: NF270 > TMN20H-400 > VNF2-8040 ≈ NF90. The former implies that the membrane surface of NF90 contains more positive charge while the membrane surface of VNF2-8040 has less unreacted amine groups, leading to the least positive charge content. The latter indicates that membrane NF270 possess more negative surface charge while unreacted acid chloride groups on the membrane surface of VNF2-8040 and NF90 are the least. 

The isoelectric point (IEP) of NF90, NF270, VNF2-8040 and TMN20H-400 is 4.25, 3.18, 4.26 and 3.92, respectively. In general, the membranes possess a positive charge at pH values below the IEP, while the membranes become negatively charged at pH values above the IEP. Hence, most current transport models used to investigate rejection properties of NF membranes assume that the membrane charge is homogeneously distributed over the pore surface [10,31,54]. Otherwise stated, the membrane volume charge density is considered to be independent of the axial position inside pores and thus, the dependence of the membrane charge with the local ion concentration is disregarded. However, this assumption may be wrong for many NF membranes. From the view of thin polyamide films synthesis by interfacial polymerization, it can be considered that the active layers of polyamide membranes are heterogeneously charged and formed of a negatively charged outer part sitting on top of an inner part containing a positive charge density [9]. This amphoteric characteristic of the membrane active layer, which are distributed in an uneven style, has been proved to have significantly influence on the separation mechanism (i.e., steric, electrostatic and dielectric exclusions) that governed the retention performance of the membranes by our previous work [21,22,55,56,57]. 

**Figure 5:**
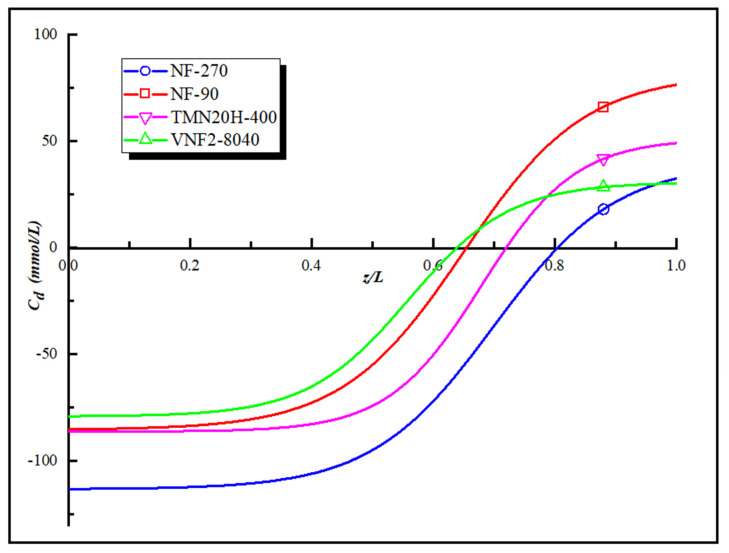
The “model” fixed charge distribution of the four polyamide membranes.

Figure 5 is the “model” fixed charge distribution of the four polyamide membranes obtained by applying the Equations (5) and (6) and their corresponding expressions are given in the Supporting Information. As shown in Figure 5, all the membranes investigated possess a maximum negative charge at the pore entrance and a maximum positive charge at the exit of pore. We obtain the order of the charge concentration at the pore entrance and pore exit of the four NF membranes as follow NF270 > TMN20H-400 > NF90 > VNF2-8040 and NF90 > TMN20H-400 > VNF2-8040 > NF270, respectively. The deviation between the fitted curve and the total fixed charge distribution converted by zeta potential is less than 5%. However, there is a difference between the total fixed charge distribution and the value of zeta potential that comes from the influence of the membrane pore size according to the Equation (5). The high zeta potential value can only explain the average charge concentration in the membrane pores, but with the change of the pore size, the fixed charge concentration on the membrane surface will inevitably change in the opposite trend, leading to changes in the electrostatic effect. 

### 3.6. Separation Performance and Mechanism of Sulfadiazine

**Figure 6:**
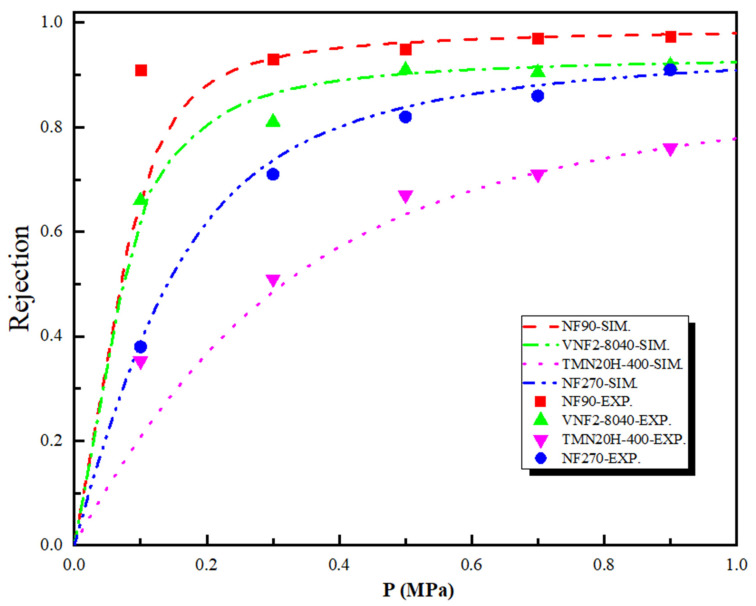
Theoretical and experimental rejection rate of the sulfadiazine (SDZ) as a function of the pressure difference across the nanopore for the four polyamide membranes.

Figure 6 shows the theoretical and experimental rejection rate of the SDZ as a function of the pressure difference across the nanopore for the four polyamide membranes. As shown in Figure 6, the SDZ rejection rate obtained by SEDE model has similar qualitative results with that of experiments and follows the sequence: $R_{NF90}>R_{VNF2-8040}>R_{NF270}>R_{TMN20H-400}$. Note that although the size of SDZ ($r_{stokes}=0.4\;\mathrm{nm}$) is larger than the pore size of NF90 ($r_{p}$ = 0.34 nm) and VNF2-8040 ($r_{p}$ = 0.34 nm), the rejection rate for both membranes is lower than 100%. Experimentally, this is mainly because (i) these four kinds of membranes are polyamide material, so the radius of membrane pore is not uniform. This implies that the size of some pores of the membrane will be larger than their average radius, thus reducing the retention rate of SDZ and (ii) the membrane pore will expand to different degrees, which will also lead to the enlargement of the pore size with the progress of filtration experiment. We observe that although the rejection trend is in line with the order of the radius of the membrane pore (i.e., a smaller pore size leads to a larger rejection rate), electrostatic and dielectric effects contribute most to the separation performance due to the charge of sulfadiazine. This can be explained by comparing the result of NF90 with VNF2-8040 which have almost the same membrane pore sizes. The retention rate of NF90 is much higher than that of VNF as the pressure difference is greater than 0.2 MPa. Although VNF2-8040 membrane has a higher average fixed charge concentration ($C_{Loc}^{ave}=-33.0\;\mathrm{mmol}/L$) than that of NF90 ($C_{Loc}^{ave}=-25.8\;\mathrm{mmol}/L$), NF90 possesses a higher concentration of negative charge at the entrance of pore and more negative charge areas inside the nanopore. Our previous studies have indicated that membrane separation properties and the rejection rate are governed by the charge concentration at the pore entrance at high driving forces whatever the variation of the fixed charge distribution inside the nanopore [55,56,57]. This conclusion is also consistent with the case of NF270 and TMN20H-400, which have similar pore radius, but the rejection rate of NF270 is significantly higher than that of TMN20H-400. However, the separation mechanism mentioned above is not suitable for nanofiltration membranes with different pore sizes, because different separation mechanisms are in competition with each other for the retention effect. For example, although the fully linear TMN20H-400 membrane possesses an average fixed charge concentration −33.0 mmol/L that higher than that of NF90 and same charge density at the pore entrance, the rejection rate of NF90 is 30% higher than TMN20H-400 at 0.5 MPa. The reason is that the stokes radius of SDZ ($r_{s}=0.4\;\mathrm{nm}$) is larger than the pore size of NF90 membrane ($r_{p}=0.34\;\mathrm{nm}$) and smaller than that of TMN20H-400 membrane ($r_{p}=0.44\;\mathrm{nm}$), so steric effect plays a decisive role in the process of separation. It should be emphasized that in the above exposition, we mainly compare the different membrane rejection efficiency through the steric and electrostatic effect. This is mainly because the dielectric effect itself has no direct influence on the steric and electrostatic exclusion [21,22], but it plays a significant role in improving the retention rate. To this end, we carried out another simulation of regardless of dielectric effect (results not shown in this work) to calculate the rejection rate of SDZ with the four membranes investigated. As expected, the rejection rate based on the both steric and electrostatic effect is significantly lower than that of the experiment. Moreover, dielectric effects do not distinguish, from a qualitative point of view, between co-ions and counterions (“counterions” is defined relative to the symbol of the charge density at the pore entrance) since they are dominated by the square of the ion charge [15,58].

**Figure 7:**
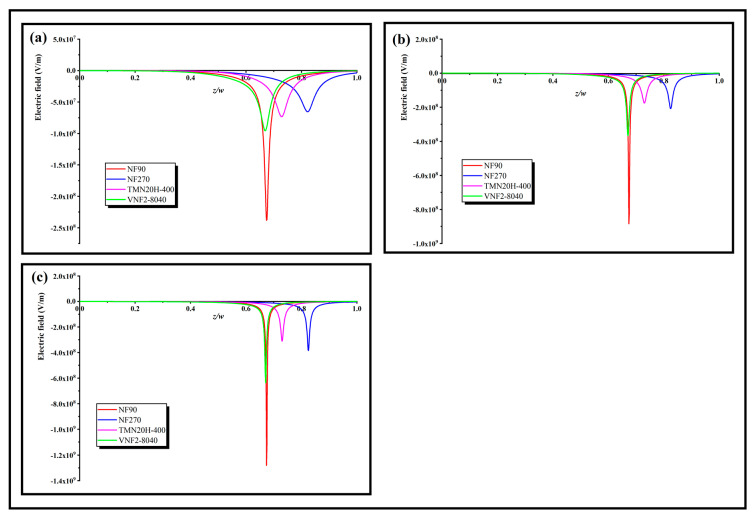
Local electric field inside the nanopore at a (**a**) high volume flux ($J_{v}=6\times 10^{-4}\;m/s$), (**b**) middle volume flux ($J_{v}=3\times 10^{-4}\;m/s$) and (**c**) low volume flux ($J_{v}=0.6\times 10^{-4}\;m/s$).

In order to further explore the role of these three separation mechanisms on the rejection effect, a rational physical explanation of the separation ability can be obtained by inquiring the electric field appearing through the nanopores, which can be calculated at any given volume flux by applying Equation (7). Figure 7a–c shows the local electric field inside the nanopore at low, middle and high volume flux for different membranes, respectively. As can be seen, the electric field is negative for all the membranes and its strength enhances trenchantly in the conversion region, which serves as a screen that tremendously blocks the transmit of counterions through the back half of the nanopore. Since the electric field is forcefully negative in the screen, it impels the cations toward to the opposite direction of the volume flux. The sunken pit of the electric field presented in Figure 7 stems from the sudden change of the fixed charge distribution in the back half zone of the pore. The larger the obliquity of the fixed charge distribution, the deeper the pit of the electric field, and the SDZ- transmission could be entirely limited (i.e., $R_{i}\to 100\%$) by a strong enough electric field. This character derives from the electric field that occurred through the nanopore in order to keep the electroneutrality of the system (Equation (4)). For a relative higher volume flow ($J_{v}=6\times 10^{-4}\;m/s$), the intensity of the electric field is almost an order of magnitude higher than that of the low volume flow ($J_{v}=0.6\times 10^{-4}\;m/s$). Physically, this can be explained by an algebraic sum of two terms in the right-hand side of expression of electric field (Equation (7)). The first term is in Equation (7) lies on the volume flux and the second term depends on the both the variation degree of fixed charge density along the nanopore and volume flux through $\langle c_{i}\left(z\right)\rangle$. As the volume flows increases, both terms in the right-hand side of Equation (7) enhances the electric field through the nanopores and thus increase the rejection rate. In addition, we note that the depth of the electric field pit of NF270 is less than that of TMN20H-400 at low flux, because of the smaller average electric field density of NF270 relative to TMN20H-400. With the increase of volume flux, the depth of the electric field pit of NF270 increases gradually and exceeds that of TMN20H-400, which is probably due to the fact that the influence of fixed charge concentration of NF270 on the electric field is greater than that of TMN20H-400 by reason of the smaller pore size of NF270 relative to the TMN20H-400. 

**Figure 8:**
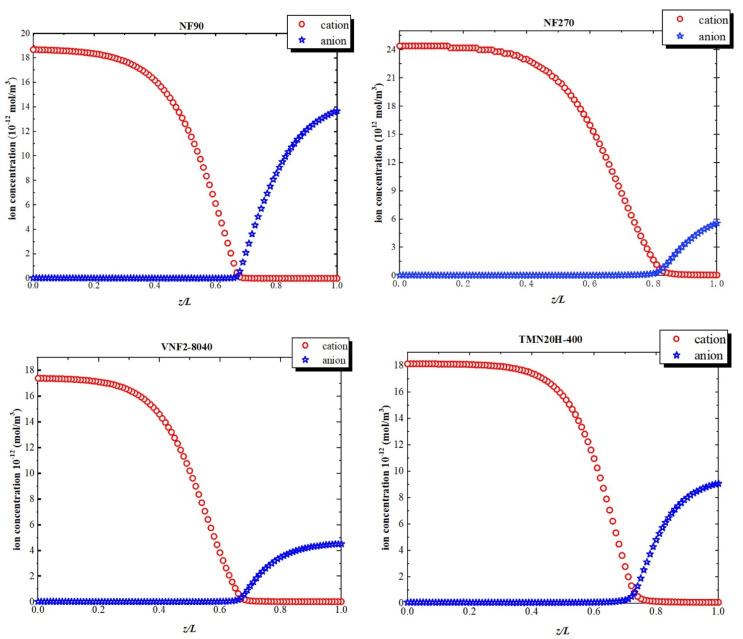
Ion concentrations inside the nanopores at a high-volume flux ($J_{v}=6\times 10^{-4}\;m/s$).

Figure 8 shows the ion concentration profiles inside the nanopores at a high volume flux ($J_{v}=6\times 10^{-4}\;m/s$). At the pore entrance, the concentration of cation is higher than that of anion due to the negative fixed charge at the pore inlet. As the variation of electric field within the pore, the concentration of the cations declines remarkably within the transition due to the sudden drop in the negative electric field. Similarly, at the exit just inside the nanopore, the concentration of anion augment on account of the positive fixed charge at back half of the nanochannel. This phenomenon of the solute concentration variation is attributed to electric field arisen from the fixed charge distribution inside the nanopore originates in the electric field that arises through pores so that the system can maintain the electroneutrality condition.

## 4. Conclusions

Sulfadiazine rejection properties of nanofiltration polyamide membranes have been investigated with a complete steric, electrostatic, and dielectric mass transfer model. It has been shown that NF90 and VNF2-8040 can be regard as fully crossed-linked polyamide and membrane TMN20H-400 is a fully linear material and NF270 is a semi aromatic chemistry with highly crossed-link density. 

According to the zeta potential analysis, all the membranes investigated possess a maximum negative charge at the pore entrance and a maximum positive charge at the exit of pore. We obtain the order of the charge concentration at the pore entrance and pore exit of the four NF membranes as follow NF270 > TMN20H-400 > NF90 > VNF2-8040 and NF90 > TMN20H-400 > VNF2-8040 > NF270, respectively.

Finally, experimental separation performance of SDZ has been compared by calculation of model SEDE. It has been shown that the SDZ rejection rate obtained by SEDE model has similar qualitative results with that of experiments and follows the sequence: $R_{NF90}>R_{VNF2-8040}>R_{NF270}>R_{TMN20H-400}$. In order to explore the role of the separation mechanisms on the rejection effect, a rational physical explanation of the separation ability is obtained by inquiring the electric field appearing through the nanopores. As can be seen, the electric field is negative for all the membranes and its strength enhances trenchantly in the conversion region, which serves as a screen that tremendously blocks the transmit of counterions through the back half of the nanopore.

## Figures and Tables

**Table 1:** Specification of investigated polyamide nanofiltration membranes.

Membrane	NF90	NF270	VNF2-8040	TMN20H-400
Surface material	Phenylenediamine and benzentricarbonyl trichloride	Semi-aromatic piperazine-based polyamide	Polyamide	Polyamide
The molecular weight cut off (MWCO) (Da)	200	150~200	<300	200
pH range	2~12 ^a^	3~10 ^a^	2~12 ^b^	2~11 ^c^
Maximum operation pressure (MPa)	0.48~4.41 ^a^	0.69~4.14 ^a^	0.69~4.14 ^b^	0.69~2.5 ^c^
Temperature resistance (°C)	5~45 ^a^	5~45 ^a^	5~45 ^b^	5~45 ^c^
MgSO_4_ rejection (%)	≥97 ^a^	85~95 ^a^	≥96 ^b^	≥97 ^c^
NaCl rejection (%)	85~90 ^d^	40 ^d^	90~98 ^b^	50 ^c^

^a^ Data from [24]. ^b^ According to the manufacturers. ^c^ According to the manufacturers. ^d^ Data from [25].

**Table 2:** Physico-chemical properties, percent speciation as a function of pH and ionization equilibria [27] of Sulfadiazine.

Compound	Sulfadiazine (SDZ)
Formula	C_10_H_10_N_4_O_2_S
Chemical Structure	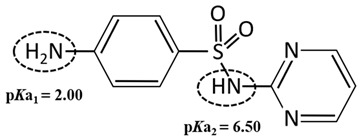
MW	250.28 g mol^−1^
pKa	2.00/6.50 ^a^
log Kow	−0.09 ^a^
Water solubility	77 mg·L^−1^ (25 °C) ^b^
Diffusion coefficient	0.605 × 10^−9^ m^2^s^−1^
Stokes radius	0.40 nm
Speciation	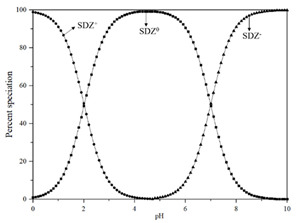
Ionization equilibria	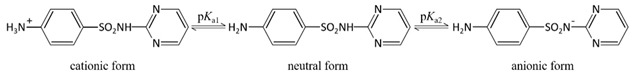

^a^ Data from [28]. ^b^ Data from [29].

**Table 3:** Molecular weight, diffusivity, and stokes radius of dioxane, erythritol and xylose.

Organic Tracer	Molecular Weight (g/mol)	Diffusivity (10^−10^ m^2^/s)	Stokes Radius (nm)
dioxane	88	9.1	0.234
erythritol	120	8.1	0.263
xylose	150	7.4	0.290

**Table 4:** Average pore radius estimations and their corresponding dielectric constants.

Organic Tracer	$r_{s}\;(\mathrm{nm})$	$\lambda \;=\;r_{s}/r_{p}$	$r_{p}\;(\mathrm{nm})$	$\varepsilon_{p}$
NF90				
dioxane	0.234	0.691	0.34	
erythritol	0.263	0.790	0.33	
xylose	0.290	0.820	0.35	
average			0.34	33.4
NF270				
dioxane	0.234	0.509	0.46	
erythritol	0.263	0.584	0.45	
xylose	0.290	0.829	0.35	
average			0.42	35.7
VNF2-8040				
dioxane	0.234	0.616	0.38	
erythritol	0.263	0.731	0.36	
xylose	0.290	0.935	0.31	
average			0.35	33.7
TMN20H-400				
dioxane	0.234	0.498	0.47	
erythritol	0.263	0.598	0.44	
xylose	0.290	0.707	0.41	
average			0.44	36.4

## Data Availability

Not applicable.

## Supplementary Materials

The following are available online at https://www.mdpi.com/2077-0375/11/2/104/s1.
