# Supplementary material for: Removal of Sulfadiazine by Polyamide Nanofiltration Membranes: Measurement, Modeling, and Mechanisms
Source: Membranes (Basel). 2021 Feb 2;11(2):104. doi: 10.3390/membranes11020104 (PMC7912794; doi:10.3390/membranes11020104)
Supplement: Supplementary file 1 [file membranes-11-00104-s001.pdf]

Supporting Information

Removal of Sulfadiazine by Polyamide Nanofiltration Membranes: Measurement, Modeling, and Mechanisms

Haochen Zhu [1,2,*], Bo Hu [1,2] and Fengrui Yang [1,2]

[1] State Key Laboratory of Pollution Control and Resources Reuse, Key Laboratory of Yangtze River Water Environment, Ministry of Education, College of Environmental Science and Engineering, Tongji University, 1239 Siping Rd., Shanghai 200092, China
[2] Shanghai Institute of Pollution Control and Ecological Security, Shanghai 200092, P. R. China

* Corresponding author, haochen_zhu@tongji.edu.cn

The "model" fixed charge distribution expressions:

Equation S1. NF90:

$$C_{Loc}(\overline{x}) = -83.4\left(-1 + \frac{2}{1 + \exp\left(10(\overline{x} - 0.65)\right)}\right) - 2.1 \quad (1)$$

Equation S2. NF270:

$$C_{Loc}(\overline{x}) = -76.5\left(-1 + \frac{2}{1 + \exp\left(10(\overline{x} - 0.7)\right)}\right) - 36.8 \quad (2)$$

Equation S3. VNF2-8040:

$$C_{Loc}(\overline{x}) = -55\left(-1 + \frac{2}{1 + \exp\left(12(\overline{x} - 0.56)\right)}\right) - 24.3 \quad (3)$$

Equation S4. TMN20H-400:

$$C_{Loc}(\overline{x}) = -68.8\left(-1 + \frac{2}{1 + \exp\left(13(\overline{x} - 0.68)\right)}\right) - 17.6 \quad (4)$$
